# Research progress on the regulatory mechanism of integrin‐mediated mechanical stress in cells involved in bone metabolism

**DOI:** 10.1111/jcmm.18183

**Published:** 2024-03-20

**Authors:** Li Yang, Hong Chen, Chanchan Yang, Zhengqi Hu, Zhiliang Jiang, Shengzi Meng, Rong Liu, Lan Huang, Kun Yang

**Affiliations:** ^1^ Department of Periodontology, Hospital of Stomatology Zunyi Medical University Zunyi China

**Keywords:** bone metabolism, cell signalling pathway, integrin, mechanical stress, tissue engineering

## Abstract

Mechanical stress is an internal force between various parts of an object that resists external factors and effects that cause an object to deform, and mechanical stress is essential for various tissues that are constantly subjected to mechanical loads to function normally. Integrins are a class of transmembrane heterodimeric glycoprotein receptors that are important target proteins for the action of mechanical stress stimuli on cells and can convert extracellular physical and mechanical signals into intracellular bioelectrical signals, thereby regulating osteogenesis and osteolysis. Integrins play a bidirectional regulatory role in bone metabolism. In this paper, relevant literature published in recent years is reviewed and summarized. The characteristics of integrins and mechanical stress are introduced, as well as the mechanisms underlying responses of integrin to mechanical stress stimulation. The paper focuses on integrin‐mediated mechanical stress in different cells involved in bone metabolism and its associated signalling mechanisms. The purpose of this review is to provide a theoretical basis for the application of integrin‐mediated mechanical stress to the field of bone tissue repair and regeneration.

## 1 INTRODUCTION

Mechanical stress refers to internal forces between various parts of an object that resist external effects when an external stimuli (such as temperature, humidity, and physical force) act on the object and cause the object to deform.^1^ Integrins are a class of transmembrane receptors that are ubiquitously present on the surface of vertebrate cells; these receptors mainly mediate the mutual recognition and adhesion between multiple cells and between cells and the extracellular matrix (ECM) and link the external and internal structures of cells.^2^ Integrins affinity for extracellular ligands is regulated through cytoplasmic proteins, and these receptors aggregate upon ligand binding,^3^ resulting in increased mechanical linkages between cells and the ECM, the rearrangement of the cellular framework (CF), and further signalling transduction.^4^ Bone metabolism is a dynamic process maintained by osteoblasts and osteoclasts and is regulated by mechanical stress, chemical/hormonal molecular signals and bone tissue damage.^5^ An increasing number of studies have shown that mechanical stress is crucial in regulating bone tissue function and an indispensable element in bone tissue engineering. Bone tissue can be damaged to varying degrees by developmental defects, trauma or infectious diseases; thus, effectively and stably restoring the original local tissue morphology and organ function has become a popular topic in bone tissue engineering research in recent years.

Wolff's law^6^ links the strain induced by mechanical stress with bone metabolism at a theoretical level. In recent years, scholars have found that integrin‐mediated mechanical stress plays an important role in the regulation of bone metabolism.^7^, ^8^, ^9^ When external mechanical stimuli act on cells, integrins located on the cell surface can sense the stimuli and bind to ligands in the ECM, thereby converting external mechanical stimuli into bioelectrical cell stimulation. Integrins also play important roles in tissue development and homeostasis, and integrin dysregulation is often associated with diseases.^10^, ^11^ The mechanism by which mechanical stress‐mediated stimulation regulates bone metabolism involves promoting the proliferation and differentiation of osteoblasts,^12^ inhibiting osteoclastogenesis^13^ and promoting angiogenesis.^14^

Bone metabolism involves the formation and resorption of bone tissue. Osteoblasts and osteoclasts are the main cells involved, and bone marrow mesenchymal stem cells (MSCs), osteocytes and chondrocytes also regulate the growth of osteoblasts and osteoclasts. Many studies have shown that when various cells involved in the regulation of bone metabolism (such as osteoblasts, osteoclasts, chondrocytes and MSCs) are exposed to external mechanical stimuli, the cells can convert external mechanical stimuli into intracellular bioelectrical stimuli through integrins on their surfaces^8^, ^15^, ^16^ and activate related intracellular signalling pathways, thereby regulating the formation and degradation of bone tissue.

**FIGURE 1:**
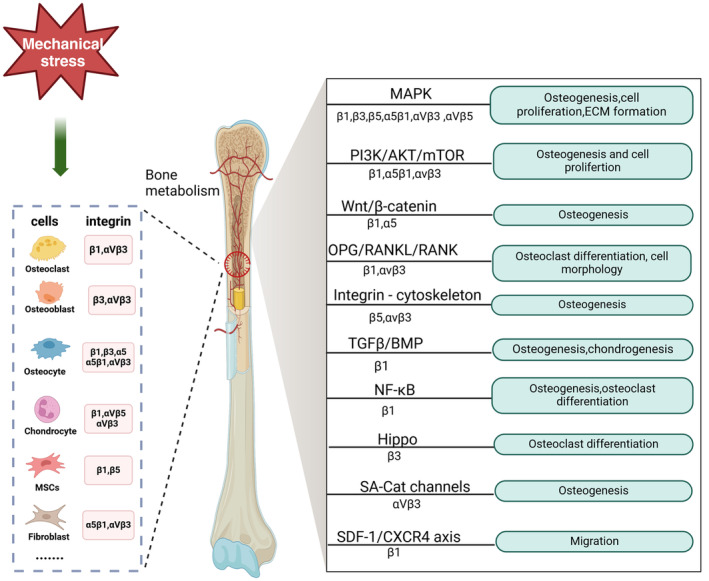
Summary of related pathways involved in integrin‐mediated mechanical stress regulation of bone metabolism. Figure was created with Biorender.com.

This paper reviews progress of research on integrin‐mediated mechanical stress in bone metabolism; introduces the characteristics of integrins and mechanical stress and the mechanisms underlying responses to integrin‐mediated mechanical stress; and focuses on the signal transduction mechanism underlying integrin‐mediated mechanical stress in different cells involved in bone metabolism. Based on the above discussions, the research and development trends of integrin‐mediated mechanical stress in the field of bone tissue regeneration and possible future development directions are discussed. The overall aim is, to provide a theoretical basis for the application of integrin‐mediated mechanical stress to the field of bone tissue engineering repair and regeneration (Figure 1).

## 2 CONCLUSION

### 2.1 The mechanical microenvironment encompassing the cells engaged in bone metabolism

Bone metabolism mainly depends on the activity of osteoblasts and osteoclasts. Osteoblasts are responsible for the formation of new bone tissue, and osteoclasts are responsible for the degradation and absorption of old or damaged bone tissue. When the two processes are synergetic, bone resorption and deposition can occur in response to mechanical stress stimuli.^17^ In addition to osteoblasts and osteoclasts, the relevant cells involved in bone metabolism include MSCs, osteocytes, chondrocytes and other cells, such as fibroblasts, macrophages and bone lining cells. MSCs are a type of pluripotent stem cells, which characteristics common to stem cells, that is, the ability to perform self‐renewal and pluripotent differentiation.^18^ Osteocytes are structure cells that support bone, are found in bone lacunae and can simultaneously regulate the activity of osteoblasts and osteoclasts.^19^ Interstitial fluid flow is the main stress‐related factor that transmits mechanical stimuli to bone cells. Studies^20^ have indicated that osteocytes are subjected to hydrostatic pressure even in the absence of macroscopic strain, when considering physiologically relevant levels and frequencies, whether it is applied unidirectionally or as a feature of fluid statics (the pressure exerted by a stationary fluid). The intensity of this pressure has been proven to be significantly potent in activating various biological cells, osteocytes included.

Chondrocytes are the main type of chondrocytes in the cartilage stroma and cartilage defects.^21^ Bone lining cells are resting osteoblasts. When the mechanical microenvironment in bone changes, some factors in the extracellular microenvironment, such as insulin growth factor‐1 (IGF‐1) and tumour necrosis factor‐α(TNF‐α), are released and can activate these cells. At the same time, as an important source of RANKL, the cells can cause preosteoclasts to fuse and differentiate into multinucleated osteoclasts by increasing RANK/ RANKL interaction, which provide the main driving force for tooth movement and osteoclastogenesis.^22^ Macrophages are common precursors of osteoclasts and bone marrow resident macrophages in bone tissue, and research has shown that macrophages regulate bone regeneration through interacting with MSCs.^23^ All these cell types in the bone microenvironment are closely related to bone homeostasis and bone diseases.

Mechanical stress refers to internal forces between various parts of an object that resist external effects when external factors (such as temperature, humidity and physical force) act on the object and cause the object to deform.^24^ Studies have shown^25^ that main types of mechanical stimulation that act upon cells include (a) tension, that is, a force that causes cells to stretch in the direction of stimulation; (b) compression, that is, a force that decreases the cell size in the direction of the force; (c) shear force, that is, the application of mechanical stimulation parallel to the cell surface; (d) hydrostatic pressure (HP), that is, the uniform application of a force to the cell, causing its volume to decrease; (e) vibration, that is, the application of oscillatory stimuli to an object; and (f) fluid shear stress (FSS), that is, a force parallel to the fluid flow on the top of a cell membrane. Furthermore, oscillatory hydrostatic pressure is recognized as a significant biomechanical stimulus that plays a crucial role in the activities of osteocytes and the process of bone remodelling. Pastrama et al.^26^ employed micro‐mechanically derived porous‐scale pressure as a regulatory factor, integrating multi‐scale poro‐micromechanics of bone with the multi‐scale model of bone cell populations involved in bone remodelling. This approach provided oscillatory fluid static pressure within the pore spaces housing bone cells (osteocytes, osteoblasts and osteoclasts). The results indicated that under physiologically relevant loading conditions, oscillatory hydrostatic pressure might be generated within the microstructure of the bone, thereby influencing the activity of bone cells and subsequently affecting the reconstruction and regeneration of bone tissue.

The type of force a cell is subjected to is closely related to the environment.^27^ When an external stimulus acts on tissue and is delivered to a cell, the cell begins the mechanotransduction process.^28^ Previous studies have shown that this process is influenced by the following main factors: (1) cell shrinkage, leading to cytoskeleton rearrangement in response to applied mechanical stimuli;^29^(2) ECM properties,^30^ such as matrix stiffness and matrix roughness of the surface; and (3) the spatiotemporal characteristics of the applied stimulus.^31^, ^32^ Previous research has indicated that mechanical loading is applied and measured based on the consideration of length scale‐related factors.^33^ Mechanical stress, whether defined as the force per unit area in a one‐dimensional analysis or through the Cauchy stress tensor in a three‐dimensional context, is highly dependent on the size of the area being measured. Consequently, the same piece of biological material may be associated with different stress levels depending on the measurements and calculations performed at various scales.

### 2.2 Structure, regulation, ligands and functions of integrins

Integrins are heterodimeric transmembrane glycoprotein receptors distributed on the cell surface.^34^ They are the most important cell adhesion molecules and signal transduction proteins in mammals. Integrins are heterodimers composed of two subunits, an α subunit (150 ~ 210 kD) and β subunit (90 ~ 110 kD). The family has a total of 18 α subunits and 8 β subunits, constituting 24 different cell membrane receptors through different α and β subunit combinations.^35^, ^36^ Each integrin subunit consists of three parts: a large extracellular domain, a small intracellular domain and a transmembrane domain.^37^ The extracellular domain can interact with the ECM to regulate cell adhesion and communication; the intracellular domain binds to intracellular regulatory proteins (such as talin and kindlin) and is responsible for signal transduction.^38^ The N‐terminus of the integrin α subunit has a domain that can bind divalent oxygen ions, and the cytoplasmic region near the membrane has a very structurally conserved FXGFFKR sequence^39^ that is mainly responsible for regulating integrin activity. The extracellular part of the β subunit is a cysteine‐rich region composed of amino acids containing internal disulfide bonds, and the cytoplasmic tail of some β subunits has a Thr‐Thr‐Thr (TTT) sequence, which facilitates the stable binding of ligands.^36^ In addition, the cytoplasmic tails of the β1, β2 and β3 subunits contain binding sites for cytoskeleton‐related proteins (such as actin), which connect integrins to the cytoskeleton.

**FIGURE 2:**
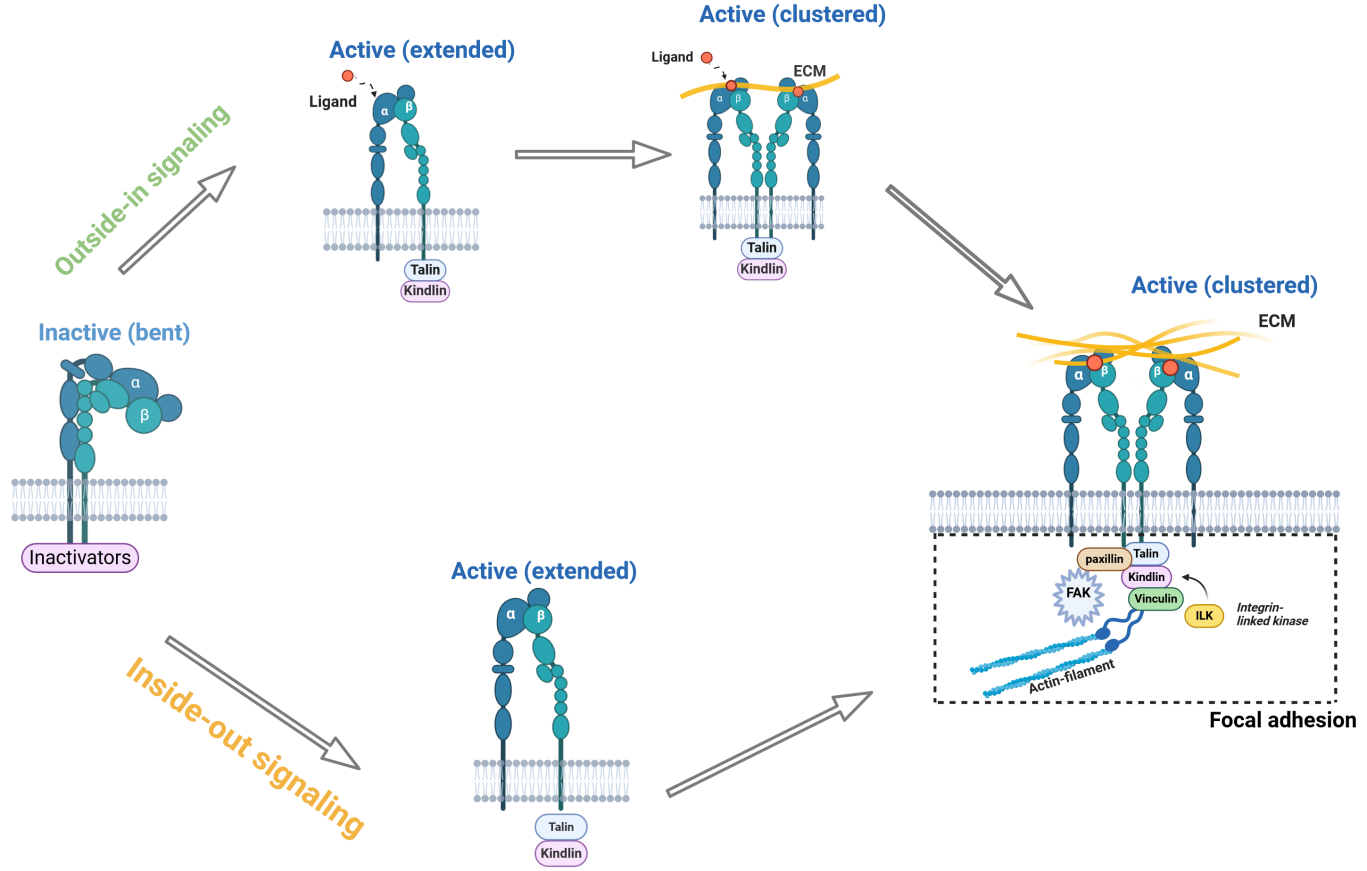
Schematic diagram showing integrin activation. Figure was created with Biorender.com.

Integrin‐mediated signal transduction, including mechanical signal transduction, is mainly achieved through the specific binding of integrins to specific ligands in the ECM.^40^ Arg‐Gly‐Asp (RGD) is the most common and important integrin ligand^41^ and is composed of arginine, glycine and aspartic acid^42^; this ligand occurs in a variety of ECM proteins, including fibronectin, vitronectin, fibrinogen, osteopontin and laminin. In addition to RGD, Ile‐Lys‐Val‐Ala‐Val (IKVAV)^43^ and Tyr‐Ile‐Gly‐Ser‐Arg (YIGSR)^44^ are laminin specific, and Pro‐His‐Ser‐Arg‐Asn (PHSRN) is fibronectin specific^45^; as a result, signal transduction can occur through integrins. As a ‘bridge’ between the inside and outside of cells, integrins can transmit transmembrane signals bidirectionally to regulate cellular function. Without a stimulus, most integrins are found in a low‐affinity folded conformation. When cells receive an activation signal, intracellular regulatory proteins, such as talin and kindlin, bind to the intracellular segment of integrin β subunits and cause the intracellular domains of integrin α and β subunits to separate, leading to conformational changes in the extracellular structural domains. In this process, integrins change from a folded non‐activated state (low affinity) to an extended activated state (high affinity), which activates integrins (inside‐out signalling). Additionally, the extracellular domain of integrins can bind to ligand proteins in the ECM, which triggers, conformational changes in integrins, causes integrins on the cell membrane surface to cluster and, allows signals to be transmitted to the nuclear membrane^46^ by FAs composed of signalling proteins, such as FAK, paxillin, talin, vinculin and integrin‐linked kinase (ILK); in addition, outside‐in signalling is simultaneously activated^47^, ^48^, ^49^ (Figure 2).

Integrins are widely expressed in animals and plants, and at least one integrin type is expressed on the surface of most cells, playing a key role in a variety of life activities. Integrins not only mediate the mutual recognition and adhesion of multiple cells and cells to the ECM but also transmit signals through the plasma membrane, thereby regulating bone metabolism, inflammation and immunity as well as the growth and development of tissues and organs.^10^, ^50^, ^51^ In addition, cells initiate apoptosis if integrin‐mediated adhesion between cells and the ECM is impaired.^52^

### 2.3 Integrin‐mediated mechanical stress and bone metabolism

Mechanical stress stimuli drive many physiological processes, including bone metabolism,^53^ sensory and motor processes,^54^, ^55^ and angiogenesis.^56^ As the first molecular receptor in cells to sense mechanical stimuli,^57^ integrins convert extracellular mechanical signals into intracellular bioelectrical signals, thereby affecting various life activities of cells. Integrins are activated under the action of mechanical stress and act as mechanoreceptors to physically connect with ligand proteins in the ECM and transmit extracellular signals to cells.^55^ Additionally, integrins are directly connected to the cytoskeleton to sense intracellular signals that alter the interaction of cells with the extracellular environment^58^ and regulate a variety of cellular functions, including bone metabolism, cell proliferation and apoptosis, angiogenesis, and ECM synthesis and degradation.^59^, ^60^, ^61^

Studies have shown that the response mechanism to integrin‐mediated mechanical stress in cells may be as follows. (1) When cells are mechanically stimulated, integrins bind to ligand proteins in the ECM, and then, integrin molecules cluster on the surface of the cell membrane. A variety of proteins (such as talin, kindlin and ILK) are recruited to form FAs.^62^ (2) FAs directly transmit the mechanical stimulation of cells to the cytoskeleton, and the deformation of the cytoskeleton causes the phosphorylation of intracellular chemical signalling molecules, such as mitogen‐activated protein kinase (MAPK), phospho‐inositol 3 kinase (PI3K) and extracellular signal‐regulated kinase 1/2 (ERK1/2),^63^, ^64^, ^65^ to undergo phosphorylation; in addition, the corresponding signalling pathways are activated, resulting in intracellular signal transduction. Numerous studies have shown that bone metabolic activity is regulated by integrin‐mediated mechanical stress. Studies have also shown that mechanical stress can lead to the redistribution of integrins on the surface of osteosarcoma cells (TE85 cells) and that pretreatment with anti‐integrin function‐blocking antibodies can inhibit the activity of mechanosensitive ion channels.^60^ A study by YAN et al. showed that mechanical stress increased the proliferation rate of MC3T3‐E1 cells and that the cell proliferation rate decreased after the specific knockout of the integrin gene.^66^ Another study showed that integrins mediate osteoblast differentiation and ECM formation and that mechanical tensile strain enhanced this differentiation and ECM formation.^67^

### 2.4 Mechanism of integrin‐mediated mechanical stress in cells involved in bone metabolism

The cell types involved in the regulation of bone metabolism facilitated by integrin‐mediated mechanical stimulation are still being explored. Cells involved in bone metabolism related to integrin‐mediated mechanical stimulation mainly include osteoblasts, osteoclasts, osteocytes, chondrocytes, MSCs and other cells, such as fibroblasts (Tables 1, 2, 3. This article summarizes the relevant literature, aiming to provide a reference for research on the precise treatment of mechanical stress‐related diseases and the optimization of bone tissue engineering under mechanical stress stimulation.

**TABLE 1:** Summary of the related mechanisms underlying integrin‐mediated mechanical stress in osteoblasts and osteoclasts involved in bone metabolism.

Cell types	Integrins	Method of treatment	Biological behaviour	Regulation	Outcome indicator	Specific mechanism	Ref.
Osteoblasts	β1	Cyclic tensile stress	Cell proliferation	Promote	Upregulate the OD value	Activation of the integrin‐ERK pathway	^63^
	β1	Cyclic stretch stress	Osteogenesis and ECM formation	Promote	Increase in ALP, Runx‐2, Col 1, OCN, OPG expression and the Ca^2+^ content, GAG and collagen in the ECM	Activation of the FA pathway	^8^
	β1	Stretching force	Osteogenesis	Promote	Increase in ALP, Runx‐2, OCN, BMP‐2 and BMP‐4 expression	Activation of the Wnt/β‐catenin signalling pathway	^73^
	αvβ3, β1	OSS	Osteogenesis and cell proliferation	Promote	Increase in c‐fos, Egr‐1 and Cox‐2 expression	Activation of P13K/Akt/mTOR/P70S6K pathway	^75^
	αvβ3	SMG	Osteogenesis	Inhibit	Decrease in ALP and Col1α expression of mRNA	Inhibition of the integrin‐IRS‐PI3K pathway	^70^
		HG	Osteogenesis	Promote	Increase in ALP and Col1 α1mRNA expression of mRNA	Activation of the integrin‐IRS‐PI3K pathway	
Osteoclasts	αvβ3	Cyclical tensile force	Osteoclast differentiation	Inhibit	Decrease in DC‐STAMP, OC‐STAMP and E‐cadherin expression	Activation of the integrin‐RANKL pathway	^78^
	β3	Low intensity electromotive force	Osteoclast differentiation	Promote	Increase NFATc1, TRAP, CTSK, MMP9 and DC‐STAMP	Activation of the integrin‐FAK‐ERK pathway	^79^
	αvβ3	Substrate stiffness	Osteoclast differentiation	Promote	Increase NFATc, Acp5, CTSK, Camk2a, Mmp9, Rela, and Traf6 and DC‐stamp expression	Activation of the integrin‐ERK‐CF pathway	^80^
	β3	Substrate stiffness	Osteoclast differentiation	Promote	Upregulation of ROCK1 and decrease in RhoA, ROCK2	Activation of integrin‐RhoA‐ROCK2‐YAP and NF‐κB pathway	^81^

**TABLE 2:** Summary of the related mechanisms underlying integrin‐mediated mechanical stress in osteocytes and chondrocytes involved in bone metabolism.

Cell types	Integrins	Method of treatment	Biological behaviour	Regulation	Outcome indicator	Specific mechanism	Rfe.
osteocytes	β1	Tibial loading model	Osteogenesis	Inhibit	Decrease in ALP, Runx‐2, Colα1,MAR,MS/BS, P1NP, SP7 expression	/	^67^
			Adipogenesis	Promote	Increase in Pparγ, Adipo, AP2 and Cebpα expression	/	
	α5β1	Shear stress	Osteogenesis	Promote	Upregulation of collagen and PGE2 expression	Activation of the P13K signalling pathway	^90^
	α5β1	FSS	Osteogenesis	Promote	Upregulation of Col1 α1 and PGE2 expression	Activation of the P13K/AKT signalling pathway	^89^
	α5	Cyclic axial loading	Osteogenesis	Promote	Increase in Cox‐2 and PGE2 expression; decrease in SOST expression	Activation of the β‐catenin signalling pathway	^91^
	αVβ3	Mechanical stress	Osteogenesis	Promote	Increase in c‐fos, IGF‐1 and Cox‐2 expression	Activation of stretch‐activated cation (SA‐Cat) channels	^92^
	αVβ3	FSS	Cell morphology	Inhibit	Decrease of the Cox‐2 and PGE2 expression	Activation of the RANKL/OPG signalling pathway	^93^
	β3	Femur three‐point bending	Osteogenesis	Promote	Decrease of p‐FAK Talin1 and p‐Talin expression	Activation of the FA pathway	^94^
	αVβ3	Laminar oscillatory fluid flow	Osteoclast differentiation	Promote	Increase of the Cox‐2 expression; decrease in the OPG expression	Activation of the RANKL/OPG signalling pathway	^97^
Chondrocytes	β1	Periodic mechanical stress	Cell proliferation, ECM formation	Promote	Increase in the ERK 1/2, RAC 1, PLCγ1 and Src phosphorylation level	Activation of the integrin β1‐Src‐PLCγ1/Rac1‐ERK 1/2 pathway	^100^
	β1	Periodic mechanical stress	Cell proliferation and ECM formation	Promote	Increase in ERK1/2 phosphorylation level, GAGs and col II production	Activation of the integrin‐ERK1/2 pathway	^7^
	β1	Periodic mechanical stress	Cell proliferation and ECM formation	Promote	Increase in CaMKII‐Thr286 and Pyk2‐Tyr402 phosphorylation level	Activation of the integrin β1‐CaMKII‐PYK 2‐ERK 1/2 pathway	^65^
	αVβ3, αVβ5	Excessive mechanical stress	Inflammation	Promote	Upregulation of IL‐1β, TNF‐α, MMP‐3 and MMP‐13 expression	Activation of the integrin‐FAK‐ERK1/2 pathway	^11^
	αVβ3	Excessive mechanical stress	Inflammation	Promote	Upregulation of Runx‐2, MMP‐9, 13 and Adamts‐5 expression	Activation of the integrin‐FAK‐ERK‐Runx‐2 pathway	^103^

**TABLE 3:** Summary of the related mechanisms underlying integrin‐mediated mechanical stress in MSCs and others involved in bone metabolism.

Cell types	Integrins	Method of treatment	Biological behaviour	Regulation	Outcome indicator	Specific mechanism	Ref.
MSCs	β1	LIPUS	Migration	Promote	Increase the migration rate	Activation of the SDF‐1/CXCR4 axis	^106^
	β1	LIPUS	Chondrogenesis	Promote	Increase in Col 2, aggrecan and Sox9 expression and decrease in Col1	Activation of the integrin‐mTOR signalling pathway	^107^
	β1	LIPUS	Osteogenesis	Promote	Increase in integrin β1, ALP and Runx‐2 expression	/	^108^
	β1	Cyclical pressure	Osteogenesis	Promote	Increase in OPN, TGF‐R1, PDGF‐α and Smad5 expression	/	^109^
	β1	FSS	Osteogenesis	Promote	Increase in ALP, Runx‐2, Col1α and OCN expression	Activation of the integrin‐FAK‐ERK1/2 pathway and the NF‐κB signalling pathway	^110^
	β5	HP	Osteogenesis	Promote	Increase in ALP, Col1, OCN and OPN expression	/	^111^
	β1	FSS	Osteogenesis	Promote	Increase in ALP, Runx‐2, OCN and OPN expression	Activation of the integrin β1‐FAK‐ERK pathway	^114^
Other cells	α5β1,αVβ3	Cyclic strain	Cell proliferation	Promote	Upregulation of cell proliferation rate	Activation of the MAPK signalling pathway	^119^
	αvβ3	CTS	Osteogenesis, cell proliferation	Promote	Increase in ALP, Runx‐2 and YAP protein expression	Activation of the integrin‐FAK‐ERK pathway and the integrin‐microfilament axis pathway	^60^

#### 2.4.1 Osteoblasts

Osteoblasts express several types of integrins, including integrin β1 (which can dimerize with α subunits, including α1, α2, α3, α4, α5, αv), β3 and β5, which have been shown to play important roles in osteogenic proliferation and differentiation.^68^, ^69^, ^70^ As mechanoreceptor cells, osteoblasts can sense and respond to a variety of mechanical stress stimuli. The MAPK pathway, which is an important mechanical signal transduction pathway, is closely related to mechanical transduction. A variety of signalling molecules in this pathway, such as ERK 1/2, c‐Jun N‐terminal kinase (JNK) and p38MAPK (p38), have been shown to facilitate a variety of important physiological/pathological processes, such as cell proliferation and differentiation, adaptation to surrounding environmental stress and inflammatory responses. Studies have shown that ERK1/2 is involved in the proliferation, differentiation and survival of different cell types, including cells involved in bone metabolism. YAN et al.^63^ applied cyclic tensile stress to MC3T3‐E1 osteoblasts and found that mechanical stimulation upregulated intracellular ERK1/2 phosphorylation and cell proliferation. After the MEK 1/2 inhibitor PD98059 was used to selectively block Raf activation of MEK 1/2, the ERK1/2 cascade was blocked, which manifested as a significant decrease in cell proliferation; as an upstream molecule of ERK1/2, MEK1/2 controlled the proliferation and differentiation of MC3T3‐E1 cells through the MAPK signalling cascade.^71^ The researchers further investigated the role of integrins in ERK activation and proliferation of MC3T3‐E1 osteoblasts, showing that the knockdown of integrin β1 reduced ERK phosphorylation levels and MC3T3‐E1 cell proliferation rates; in contrast, knockdown of integrin β5 significantly increased ERK phosphorylation and cell proliferation rates. These results suggest that mechanical strain can convert extracellular mechanical signals into intracellular biological signals through the ERK signalling pathway and that integrin β1 and β5 may play opposite roles in this process. Other investigators applied cyclic stretch stress to MC3T3‐E1 cells to clarify the relationship between β subunits of integrin in mechanical tension‐induced osteoblast differentiation and ECM formation; the results indicated that integrin β1 (but not integrin β5) mediated osteoblast differentiation and ECM formation upon mechanical tension stimulation. Notably, the simultaneous knockout of integrin β1 and β5 weakened the inhibitory effect of osteoblast differentiation and ECM formation, suggesting that integrin β5 knockout could attenuate the inhibitory effect of integrin β1 knockout on osteoblast differentiation and ECM formation.^8^ However, the exact relationship between the two in the biological activities of osteoblasts has not been elucidated, and further research is needed.

Relevant studies have shown that the Wnt/β‐catenin signalling pathway plays an important role in the process by which osteoblasts respond to mechanical stress.^72^ Mechanical stimulation can be mediated by integrins, thereby activating the Wnt/β‐catenin pathway to induce bone formation. Studies have shown^73^ that stretching force can increase the expression of integrin β1 mRNA, phosphorylated glycogen synthase kinase‐3β (GSK‐3β) and β‐catenin protein in MC3T3‐E1 cells and upregulate the expression of osteogenesis‐related genes, such as Runt‐associated transcription factor‐2 (Runx‐2), osteocalcin (OCN), bone morphogenetic protein‐2 (BMP‐2) and BMP‐4, enhancing alkaline phosphatase (ALP) activity. In contrast, GSK‐3β and β‐catenin expression was suppressed when integrin β1 in MC3T3‐E1 osteoblasts was silenced with small interfering RNA, suggesting that stretching force can promote osteoblast differentiation through integrin β1‐mediated β‐catenin signalling. In osteoblasts isolated from the calvaria or long bones of C57BL/6J (B6) mice and treated with inhibitors of the Wnt pathway (endostatin), BMP pathway (Noggin) or ER pathway (ICI182780), the proliferation of osteoblast proliferation induced by fluid shear force was blocked; thus, osteoblast mechanotransduction and insulin‐like growth factor‐1 (IGF‐1), ER, BMP and Wnt pathway‐related genes may be upregulated. However, after fluid shear‐stimulated mouse osteoblasts were treated with echistatin (integrin inhibitor) and indomethacin (Cox‐2 inhibitor), the expression levels of genes related to the above four pathways were upregulated, indicating that in the mechanotransduction mechanism of osteoblasts, FSS upregulated the expression of at least two early mechanoresponsive genes (integrin β1 and Cox‐2) and that these four pathways may be located downstream of these two genes.^74^

Additional evidence showed that the mTOR/P70S6K (P70S6 kinase) pathway downstream of PI3K/AKT is necessary for osteoblast proliferation and differentiation.^64^ LEE et al. applied oscillatory shear stress (OSS) to human osteoblast‐like MG63 cells and confirmed that OSS can mediate FAK and Shc expression by activating integrin αvβ3 and integrin β1 and cooperate with P13K to activate downstream ERK and Akt/mTOR/P70S6K; as a result, MG63 cell proliferation is induced, which increases the expression levels of bone formation‐related genes (c‐fos, Egr‐1 and Cox‐2).^75^ DAI et al.^70^ cultured mouse MC3T36OSE2‐luc osteoblasts (OSE‐3 T3) with and without IGF‐1, PI3K inhibitor (LY294002) and the combination of these drugs under simulated microgravity (SMG) or hypergravity (HG); the results showed that SMG and HG affected the expression and activity of integrin αvβ3 and the phosphorylation level of p85 and that integrin αvβ3 interacted with IGF‐1. This effect was reduced under SMG conditions and increased under HG conditions, manifesting as changes in the mRNA expression levels of ALP and type I collagen α1 chain (Col1α1); additionally, integrin αvβ3 mediated the synergistic effect of gravity and core‐binding factor α1 (CBFA1) transcriptional activity through the P13K signalling pathway to promote the osteogenic differentiation of OSE‐3 T3 cells.

#### 2.4.2 Osteoclasts

Osteoclasts function as a crucial regulator of bone as mechanosensitive cells. The differentiation of osteoclasts has been demonstrated to be influenced by mechanical stimuli. Previous research suggests that integrin αvβ3 is the most pertinent molecule in the regulation of osteolysis, and the molecule is crucial for the proliferation of osteoclast precursor cells in the formation of multinucleated cells and subsequent bone resorption.^76^ The activated αvβ3 receptors can promote osteoclast formation and adhesion to the ECM.^77^In osteoclasts, integrin αvβ3 can specifically bind to ECM proteins through cell surface RGD sequences, resulting in decreased osteoclast activity and bone resorption.^77^ Some scholars^78^ applied tension to RAW 264.7‐induced osteoclasts and found that mechanical stimulation caused by the RANKL‐NFATc1 axis could lead to the downregulation of osteoclast‐specific gene expression and fusion‐related molecules, such as dendritic cell‐specific transmembrane proteins (DC‐STAMPs) and osteoclast‐stimulating transmembrane proteins (OC‐STAMPs); additionally, the levels of E‐cadherin, integrin αv and integrin β3 mRNA decreased. Another study showed that low intensity electromotive force could increase the expression of integrin β3, which in turn activated ERK and p38 MAPK to regulate osteoclast differentiation, resulting in the high expression of osteoclastogenesis markers NFATc1, TRAP, CTSK, MMP9 and DC‐STAMP.^79^Recent studies have shown that matrix stiffness can regulate CF alignment through integrin αvβ3, thereby regulating the differentiation and function of osteoclast.^80^WANG et al. further demonstrated that matrix stiffness similar to blood vessel stiffness could simultaneously enhance preosteoclast cell‐mediated angiogenesis and bone repair through integrinβ3‐mediated RhoA‐ROCK2‐YAP and NF‐κB signalling, thereby regulating osteoclastogenesis.^81^

From a microscopic perspective, the orthodontic process is bone metabolism. Orthodontic force affects the bone metabolism level of the alveolar bone by disrupting the balance between osteoblasts and osteoclasts in the peri‐dental bone tissue, which manifests as orthodontic tooth movement (OTM).^82^ Studies have shown that mechanical stimulation can influence OTM by affecting the activity of osteoclasts and that integrins are crucial in the process of osteoclast participation. ZHANG et al.^83^, ^84^ used in situ hybridization to detect periodontitis‐affected teeth and normal teeth in rats during orthodontic movement and found that integrin β1 was strongly expressed in osteoblasts at all times of applied force in the normal and periodontitis groups. In addition, integrin β3 was mainly expressed in the periodontal ligament and alveolar bone marrow cavity of normal teeth and teeth with periodontitis in the early stage of movement; thus, integrin β1 in osteoclasts may be involved in the entire OTM process. Integrin β3 may be involved in the transformation of osteoclast precursor cells to osteoclasts. Previously, researchers^85^ integrated echistatin and RGD peptide (a drug known to interfere with bone remodelling) into ethyl vinyl acetate (ELVAX) scaffolds and then applied the scaffolds to the maxillary molars of rats during tooth movement. The topical administration of integrin inhibitors blocks osteoclast‐mediated OTM, and the main mechanism mainly involves disrupting the actin ring (a key marker of functional osteoclasts) that is specific to osteoclasts. Actin is an important protein component of the cytoskeleton and is closely related to cell proliferation, apoptosis and immune regulation.^86^

#### 2.4.3 Osteocytes

As the most abundant cells in bone, osteocytes are mainly responsible for regulating the balance between bone formation and bone resorption.^19^Osteocytes can produce RANKL, sclerostin (SOST), OPG and other biological factors, thereby regulating the balance between osteoclasts and osteoblasts to regulate bone homeostasis. Integrins β1 and β3 have been shown to be essential for bone cell mechanotransduction.^87^Integrin β1 is mainly found on the plasma membrane around the cell body and is among the integrin subunits mainly expressed by bone cells, which can bind to the α1, α2, α3, α4 and α5 subunits;^50^ integrin β3 is mainly associated with αV. Immunohistochemistry showed that osteocyte protrusions have unique integrin αVβ3 clusters in vivo, and both can interact with the surrounding ECM.^87^, ^88^Animal studies showed that the absence of integrin β1 in osteocytes could lead to severe bone mass reduction in mice, and the application of mechanical load did not increase bone formation in mice, suggesting that integrin is indispensable in the regulation of bone homeostasis by mechanical stress stimulation.^67^

In addition, studies have shown that mechanical stimulation can activate the integrin α5β1‐mediated PI3K signalling pathway in MLO‐Y4 osteocytes to open Cx43 hemichannels^89^ and promote the release of bone anabolic molecules, such as prostaglandin‐2 (PGE2), from MLO‐Y4 osteocytes, which are essential for bone formation and bone regeneration.^90^ Integrin α5 deficiency impedes the mechanical stimulation‐induced opening of Cx43 hemichannels. Conditional knockout of integrin α5 in mouse tibia cells could block the opening of Cx43 hemichannels on the tibia cell surface induced by cyclic mechanical load; however, the production and release of PGE2 were decreased, resulting in the attenuated synthesis of sclerostin (SOST) and β‐catenin, factors associated with bone catabolism.^91^

In primary bone cells, activation of integrin αVβ3 by mechanical stretch can lead to the upregulation of c‐fos, IGF‐1 and Cox‐2.^92^Blocking integrin αvβ3 using an antagonist disrupted the morphology of MLO‐Y4 bone cells; as a result, the spreading area and process retraction were reduced, the level of Cox‐2 expression was decreased and the release of PGE2 under fluid shear stress was abolished.^93^Animal experiments showed that knocking down integrin β3 under mechanical stimulation could affect the FA signalling pathway in bone cells; as a result, the expression levels of phosphorylated focal adhesion kinase (p‐FAK) and phosphorylated Talin 1 (p‐Talin) proteins related to this pathway were reduced, reducing long bone formation.^94^ Previous studies have suggested that oestrogen acts as a regulator to maintain bone metabolic balance,^95^ and enhance the response of bone cells to mechanical stress.^96^GEOGHEGAN et al.^97^ cultured oestrogen‐deficient MLO‐Y4 osteocytes under the action of laminar oscillatory fluid flow and found that oestrogen deficiency led to a smaller focal adhesion area and reduced αvβ3 localization at focal adhesion sites; as a result, the RANK1/OPG ratio increased, which promoted osteoclastogenesis. At the same time, oestrogen withdrawal can inhibit the expression of RANK1/OPG in bone cells, thereby affecting osteoclastogenesis. These results suggest that osteoclastopenia caused by mechanical stimulation can also affect osteoclastogenesis through paracrine signals.

#### 2.4.4 Chondrocytes

Integrins do not exhibit kinase activity, and they transmit mechanical signals as biochemical signals through integrin‐related signal kinases, such as Src and ERK1/2.^75^ Activation of integrin β1, which is mainly located on the membrane of chondrocytes, can accelerate the differentiation and maturation of these cells by regulating ECM synthesis, thereby promoting chondrogenesis and remodelling.^98^, ^99^ Related studies have shown that mechanical stress‐induced chondrocyte proliferation and matrix synthesis are mainly mediated by the integrin β1‐ERK1/2 signalling cascade. After cyclic mechanical stress was applied to rat chondrocytes, mechanical stress activated two signalling pathways that involved ERK1/2, that is, a Rac1‐dependent pathway and a PLCγ1‐dependent pathway, which were both dependent on Src activation. Integrin β1 was shown to link cyclic mechanical stress stimuli with Src‐ERK1/2 signalling and cause, them to converge into a mitogenic cascade in chondrocytes, promoting chondrocyte proliferation and matrix synthesis.^100^ Notably, in that study, the phosphorylation levels of activated Src, PLCγ1, Rac1, ERK1/2 and Rac1 were attenuated when chondrocytes were pretreated with an anti‐integrin β1 function‐blocking antibody. Thus, despite the presence of integrin β1, other integrin‐related kinases and MAPKs could still transmit mechanical signals. The upregulation of integrin β1 does not affect the cellular response of or ERK1/2 phosphorylation in chondrocytes when cultured under static conditions. Cyclic mechanical stress stimulation combined with integrin β1 upregulation further promotes chondrocyte proliferation and matrix synthesis, increases ERK1/2 phosphorylation levels in chondrocyte monolayer cultures and promotes the accumulation of glycosaminoglycans (GAGs) and type II collagen in chondrocyte 3D cultures.^7^ Cyclic mechanical stress may be a key factor that improves the quality of chondrogenesis and the upregulation of integrin β1 may amplify this effect; a possible explanation is that the number of mechanoreceptors increases, which allows cells to receive more mechanical stimulation. Researchers^65^ have applied cyclic mechanical stress to chondrocytes to explore the role of the CaMKII‐Pyk2 signalling pathway in chondrocyte proliferation and matrix synthesis. The results showed that mechanical stress stimulation significantly enhanced the phosphorylation of Pyk2 at Tyr402 and of CaMKII at Thr286. After researchers silenced Pyk2 and CaMKII expression with an inhibitor or shRNA, chondrocyte proliferation and matrix synthesis were attenuated, suggesting that integrin β1 can mediate cyclic stress to promote chondrocyte proliferation and matrix synthesis through the CaMKII‐Pyk2‐ERK1/2 signalling cascade.

Chondrocytes continuously receive external stimuli and regulate bone remodelling through bone metabolic homeostasis. Disruption of the bone metabolic balance can lead to bone metabolic diseases, such as osteoarthritis (OA) and osteoporosis.^101^, ^102^ The effects of mechanical stress on chondrocyte inflammation and related cellular pathways have been extensively studied. Researchers^11^ have found that cilengitide (integrin receptor antagonist) inhibits the expression of inflammation‐related genes such as IL‐1β, TNF‐α, MMP‐3 and MMP‐13 induced by excessive mechanical stress (10% elongation rate, 0.5 Hz and 3 h) and suppresses the phosphorylation levels of integrin downstream‐related molecules such as FAK, ERK, JNK and p38; these results suggest that excessive mechanical stress can activate integrins on the surface of chondrocytes and upregulate the expression of inflammatory‐related factors through the phosphorylation of FAK and MAPKs, thereby inducing cartilage inflammation. Through histological and proteomic analyses of osteoarthritic cartilage in a destabilized medial meniscus rat model and in vitro findings, SONG et al.^103^ demonstrated that excessive mechanical stress (15 V, 2 Hz) led to significantly increased integrin αVβ3 expression, enhanced the phosphorylation of downstream signalling molecules, such as FAK and ERK, and upregulated the expression of inflammation‐related proteins, such as MMP‐9, 13 and Adamts‐5; the inhibition of integrin αVβ3 attenuated chondrocyte inflammation induced by excessive mechanical stress in vivo and in vitro.

#### 2.4.5 MSCs

A number of studies have shown that a variety of mechanical stress stimuli can drive the proliferation and differentiation of BMSCs. FSS can upregulate the expression of bone markers BMP‐2, BSP and OPN in MSCs.^104^ Short‐term fluid flow stimulation could promote the expression of Cox‐2, OPN and Runx‐2 in early osteogenesis of MSCs, while long‐term stimulation increased the formation of collagen and matrix in the late stage.^105^ Several studies have shown that integrins play an important role in mechanical stimulation on bone metabolism of MSCs. Studies have shown that LIPUS not only promotes BMSCs migration through integrin β1,^106^ but also promotes chondrosarcoma differentiation of BMSCs through integrin β1 and its downstream mTOR pathway.^107^ In addition, LIPUS can also enhance the proliferation of human periodontal ligament stem cells (hPDLSCs), promote the secretion of OCN, enhance the activity of ALP, up‐regulate the expression of integrin β1 and Runx‐2, and promote the formation of mineralized nodules.^108^

Some researchers have used DNA array technology to study the effect of 24 h mechanical stress cyclic loading on the gene expression of human bone marrow stromal cells (hBMSCs). The results showed that after stress stimulation, the expression levels of genes encoding matrix molecules, receptors and growth factors increased, which significantly increased OPN, integrin β1, transforming growth factor receptor 1 (TGF‐R1) and Smad5 expression.^109^ Thus, short‐term mechanical stimulation may be involved in regulating the osteogenic differentiation of hBMSCs through integrin β1. Another study showed that under FSS, integrin β1 in hBMSCs not only promoted the formation of hBMSCs by activating FAK and its downstream molecules ERK1/2 to upregulate the expression of osteogenesis‐related genes, such as ALP, OCN, Runx‐2 and Col 1α, but also activated the NF‐κB pathway through ERK1/2 phosphorylation feedback, thereby upregulating its own expression.^110^ In addition, HUANG et al. found that HP enhanced the cell viability of hBMSCs seeded on hydroxyapatite (HA) scaffolds and promoted their osteogenic differentiation, which increased the expression of osteogenic genes, such as OCN, OPN, Col1 and ALP. Furthermore, the expression of integrin β5 mRNA increased in HP‐stimulated hBMSCs, an effect closely related to the expression of OCN, Col1 and CBFA1; thus, HP may promote the osteogenic differentiation of hBMSCs by activating integrin β5.^111^

The coculture of human umbilical vein endothelial cells (HUVECs) and hBMSCs has been studied by many researchers. Compared with culturing hBMSCs, the coculture of hBMSCs and HUVECs at a 1:1 ratio under static conditions can enhance the osteogenic differentiation of hBMSCs.^112^ DAHLIN et al. further demonstrated that in this coculture system, fluid perfusion under mechanical stress enhanced the early osteogenesis of hBMSCs.^113^ JIANG et al.^114^ cocultured rat BMSCs with HUVECs and applied FSS and found that FSS stimulation upregulated the mRNA levels of ALP, Runx‐2 and OCN in BMSCs and caused OCN and OPN protein expression to increase. Thus, the coculture of BMSCs and HUVECs may have synergized with FSS to promote the osteogenic differentiation of BMSCs, which was mediated through the integrin β1‐FAK‐ERK signalling pathway. Optimizing the mechanical conditions in coculture systems, selecting the appropriate coculture and clarifying the specific transduction mechanism will be important goals of future research on bone tissue engineering.

#### 2.4.6 Other cells

Fibroblasts are a common adherent cell type in the mesenchymal matrix^115^ that have MSC markers similar to MSCs and can differentiate towards osteogenesis, lipogenesis and chondrogenesis.^116^ Binding of fibroblasts to proteins in the ECM is regulated by integrins. The binding of fibroblasts to proteins in the ECM is regulated by integrins. A variety of integrins can be expressed in fibroblasts, such as α1, α6, α7, αv, β1 and β5.^117^ Studies have shown that integrin α5β1 has strong is highly expressed in human periodontal ligament fibroblasts,^117^ the ligand of which is fibronectin in the ECM, and the binding between the two plays an important role in hPDLF signal transduction.^118^ Some studies have found that 0.5% small cyclic mechanical stress can enhance the proliferation ability of fibroblasts and that different types of integrins exhibit different sensitivities to stress stimuli; α5β1 and αvβ3 integrins showed higher sensitivity to stress stimuli, and α1β1 and α2β1 integrins were less sensitive to mechanical stimuli,^119^ suggesting that integrin type is an important regulator of integrin mechanosensing. PENG et al.^60^ exposed fibroblasts to cyclic tensile stress (0.5 Hz, 10% strength, 2 h/d) and found that cyclic tensile stress promoted the osteogenic differentiation of fibroblasts, which leads to the upregulation of osteogenic‐related genes, such as ALP and Runx‐2; however, after the activation of integrin αvβ3 was inhibited with c(RGDyk), cellular microfilament rearrangement and ALP, Runx‐2 and YAP protein expression was downregulated, accompanied by decreased FAK phosphorylation. Thus, cyclic mechanical stress can promote the osteogenic differentiation of human fibroblasts through the integrin‐microfilament axis, and the phosphorylation of FAK and YAP is also involved.

Although a large number of studies have investigated integrin‐mediated mechanical stress regulation of bone metabolism, many factors remain unknown, such as the application of stress. Determining the type, size, duration and time threshold of mechanical stimulation is essential for performing effective and centralized mechanical treatments of bone metabolic diseases and bone tissue regeneration. Another unknown factor is which types of integrin help mediate mechanical stress to regulate bone metabolism; for example, although studies have shown that the α1 subunit of integrin can mediate mechanical stress, these studies only pertain to neural tissue^120^, ^121^; there are no reports of its application in the field of bone regeneration. Although many studies have confirmed that integrin α2 can mediate mechanical stimulation and participate in the regulation of bone metabolism, integrin α2 mainly regulates matrix hardness by changing the proportion of a certain component in the ECM.^122^, ^123^, ^124^, ^125^ No mechanical stress has been applied to the ECM around cells to stimulate integrins on the cell surface, and thereby regulate bone metabolism. Another issue is the selection of target cell types. Most related studies involved bone‐derived cells, and only a few studies employed other cells, such as fibroblasts. Some cell types have been shown to be closely related to bone metabolism, but no studies have involved integrin, such as bone lining cells and macrophages. Developing more cell types, clarifying the specific integrin types and regulatory mechanisms involved and applying the knowledge to bone tissue engineering are major challenges.

### 2.5 Nonintegrin‐mediated mechanical stress and bone metabolism

With advancements in research, knowledge on the mechanism by which cells respond to stress and adaptive strain has increased. Cells can sense extracellular mechanical stress through deformation and sense extracellular mechanical stress through mechanosensitive ion channels and integrins on the cell membrane; these structures can transmit mechanical signals directly into the cells or convert mechanical signals into chemical signals and transmit them to cells. In addition to integrins that mediate mechanical stimuli to participate in the regulation of bone metabolism, many factors can mediate mechanical stimuli to participate in the regulation of bone metabolism, such as epigenetic modifications, mechanosensitive channels, cadherins and primary cilia (Table 4).

**TABLE 4:** Summary of mechanisms pertaining to the relationship between nonintegrin‐mediated mechanical stress and bone metabolism.

Cells	Method of treatment	Biological behaviour	Regulation	Outcome indicator	Specific mechanism
MLO‐Y4 osteocytes^127^	FSS	Osteogenesis and adipogenesis	Inhibit	Increase in OCN, OPN and OSX expression; decrease in Runx‐2, PPARy, FABP 4, LPL expression	DNA methylation
hAT‐MSCs^9^	Mechanical stretching	Osteogenesis, adipogenesis and chondrogenesis	Promote	Increase in ALP activity, BGLAP, OMD, FZD4 and FRZB expression	DNA demethylation
MC3T3‐E1 cells^128^	Mechanical tensile strain	Osteogenesis	Promote	Increase in ALP, Col I OCN, BMP‐2 and BMP‐4 expression	Upregulation of the miRNAs levels
Ocy454 cells^131^	FSS	Osteogenesis	Promote	Decrease in SOST and CaMKII phosphorylation levels	Activation of the microtubule network
MC3T3‐E1 cells^133^	Low‐intensity traction force	Osteogenesis	Promote	Upregulation of the activation of NF‐κB, RANKL and NFATc1	Activation of the TRP channels
MSCs^135^	Hydrostatic pressure	Osteogenesis	Promote	Increase in ALP, Runx‐2, BMP‐2 and OSX expression	Activation of the Piezo1 channels
Chondrocytes^140^	Cyclic mechanical tension	Cell proliferation	Promote	Decrease in Col‐II α, ACAN and Sox 9 expression	Activation of the Wnt/β‐catenin signalling pathway and inhibition the cadherin‐catenin complex

Epigenetic modifications^126^ refer to chemical modifications that alter chromosomes to promote gene regulation; these modifications only change the DNA transcription process and do not change the nucleotide sequence of the DNA. Common epigenetic modifications include DNA methylation, histone modification, noncoding RNA, RNA modification and chromatin remodelling. Previous studies have shown that mechanical stress stimulation can change the state of DNA methylation so that cells can produce an appropriate epigenetic state; as a result, osteoblasts are regulated and the cells can participate in osteogenic differentiation.^127^ By applying mechanical stretching to human adipose tissue multipotential stromal cells (hAT‐MSCs), VLAIKOU et al. demonstrated that long‐term mechanical stimulation can trigger the control of DNA methylation and osteogenic differentiation of hAT‐MSCs.^9^ Another researcher used mechanical loading to induce osteogenesis in BMSCs and osteoblasts and found that miRNAs in the two changed significantly^128^; thus, miRNAs may serve as a mechanisms by which mechanical loading is mediated to regulate bone metabolism and promote bone formation.

Among the related reports on mechanosensitive channels involved in the regulation of bone metabolism, TRP and Piezo channels have been investigated; both of these channels are permeable to Ca^2+^ and can regulate bone metabolism by altering the expression of downstream genes, thereby causing changes in cell morphology and function.^129^ TRP channels are mechanosensitive channels composed of six transmembrane domains and an intracellular N terminus and C terminus.^130^ A previous study found that fluid shear force can activate the TRPV4 channel of osteocytes, increase the intracellular Ca^2+^ concentration, activate CaMK II and inhibit the secretion of sclerostin.^131^ Sclerostin is a glycoprotein secreted by osteocytes, is an inhibitor of BMP and negatively regulates bone formation.^132^ Researchers applied a low‐intensity traction force to osteoblasts and found that intracellular and extracellular Ca^2+^ concentrations are changed through TRPM3 and TRPV4 channels, resulting in enhanced NF‐κB, RANKL and NFATc1 activity and accelerated bone resorption^133^; thus, mechanical stress‐stimulated TRP channels may play a key role in bone remodelling. Piezo channels are nonselective ion channels that are expressed in a variety of cells, such as osteocytes, chondrocytes and MSCs, and respond to a variety of mechanical stress stimuli; in addition, there are two subtypes of these channels, Piezo1 and Piezo2.^134^ In an in vitro experimental study, SUGIMOTO et al. found that hydrostatic pressure and Piezo1 activator (Yoda1) promoted BMP‐2 expression and the osteogenic differentiation of MSCs and inhibited adipose differentiation; additionally, the inhibition of Piezo1 attenuated BMP‐2 expression and osteoblast differentiation.^135^

Unlike integrins, which mediate cell‐to‐ECM adhesion, cadherins mainly mediate cell‐to‐cell adhesion. Cadherins are a class of Ca^2+^ −dependent adhesion molecules and are divided into the following subtypes: E‐cadherin, N‐cadherin and P‐cadherin.^136^ Cadherins can combine with β‐catenin to form a cadherin‐catenin complex.^137^ As a sensitive mechanosensor, this complex regulates intracellular signalling pathways by linking the intracellular cytoskeleton,^138^ thereby maintaining cell differentiation and ECM protein synthesis. Previous studies have shown that the presence of E‐cadherin in the cytoplasm is necessary for the inhibition of Wnt/β‐catenin‐dependent gene expression.^139^ XU et al. found that cyclic mechanical tension stimulation can promote chondrocyte proliferation; in addition, this process was achieved by activating Wnt/β‐catenin signalling and inhibiting the physical protein interaction between E‐cadherin and β‐catenin.^140^

Furthermore, primary cilia are involved in the transduction of mechanical signals into cells. Primary cilia can sense mechanical stress through different signalling pathways, such as Hedgehog, Wnt and TGF‐β.^141^ A wide range of extracellular signals are transduced into cells to promote the regeneration of bone tissue and its surrounding blood vessels.^142^

## 3 SUMMARY AND OUTLOOK

Tissue formation and differentiation are regulated by various signals, which are triggered by biological, chemical and physical factors. Increasing data suggest that mechanical stress, which is the fourth element in bone tissue engineering after cells, scaffolds and growth factors, plays an important role in regulating many cellular functions. Integrins are effective mechanosensors due to their unique spatial arrangement. As important transmembrane receptors, the mechanical and chemical signals integrins transmit play important roles in cellular bone metabolism. Integrins are involved in many aspects of bone metabolism and exert bidirectional regulatory effects on bone metabolism.

Progress has been achieved in treatment of bone metabolic diseases through the development of various drugs and therapies that target various integrins and mediate mechanical stress in the bone regeneration process; due to the potential therapeutic and preventive value of these drugs and treatment, they will receive increasing attention. However, the application of integrin‐mediated mechanical stress in bone tissue regeneration is currently limited to cell experiments and in vitro animal experiments, and support from long‐term in vivo experimental data in large animals and clinical trial data is lacking; thus, further research is needed. Additionally, many problems remain in the application of mechanical stress in bone tissue engineering, such as the choice of stress application, the specific transduction mechanism of mechanical stimulation mediated by different integrin types, the relationship between mechanical stimulation and ECM interactions, the use of stem cells and the availability of tissue mechanotransduction therapy.

Through a series of in‐depth studies in the future, scholars will continue to deepen their knowledge on mechanical stress, further clarify the mechanisms underlying integrin‐mediated mechanical stress, explore the role of integrin‐mediated mechanical stress in bone growth, development and repair in the human body, and provide a reference for guiding bone metabolic disease treatment and sports rehabilitation in the field of bone tissue regenerative medicine.

## AUTHOR CONTRIBUTIONS

**li Yang:** Conceptualization (equal); writing – original draft (lead). **Hong Chen:** Data curation (equal). **Chanchan Yang:** Data curation (equal). **Zhengqi Hu:** Data curation (equal). **Zhiliang Jiang:** Formal analysis (equal); investigation (equal). **Shengzi Meng:** Formal analysis (equal); investigation (equal). **Rong Liu:** Formal analysis (equal); investigation (equal). **Lan Huang:** Formal analysis (equal); investigation (equal). **Kun Yang:** Funding acquisition (equal); supervision (equal); writing – review and editing (equal).

## FUNDING INFORMATION

The Science and Technology Fund of Guizhou Province (No. 2020J1Y328) (to YK); Zunyi Science and Technology Plan Project (No. Zunshikehe‐HZ(2023)79) (to YK); Guizhou Health Commission Science and Technology Fund Project (gzwkj2022‐1,692,022‐423) (to ZN); and The Basic Research Project of Department of Science and Technology of Guizhou Province (No. Qiankehe base‐ZK (2023) 535) (to ZR). Zunyi Medical University Affiliated Hospital Fund Yuan Zi (2022) 02.

## CONFLICT OF INTEREST STATEMENT

The authors confirm that there are no conflicts of interest.

## ETHICAL APPROVAL

Not applicable.

## CONSENT TO PARTICIPATE

Not applicable.

## CONSENT FOR PUBLICATION

Not applicable.

## Data Availability

Not applicable.
